# A sequence-based machine learning model for predicting antigenic distance for H3N2 influenza virus

**DOI:** 10.3389/fmicb.2024.1345794

**Published:** 2024-01-19

**Authors:** Xingyi Li, Yanyan Li, Xuequn Shang, Huihui Kong

**Affiliations:** ^1^School of Computer Science, Northwestern Polytechnical University, Xi'an, Shaanxi, China; ^2^Big Data Storage and Management MIIT Lab, Xi'an, Shaanxi, China; ^3^State Key Laboratory of Animal Disease Control and Prevention, Harbin Veterinary Research Institute, Chinese Academy of Agricultural Sciences (CAAS), Harbin, China

**Keywords:** influenza A H3N2 virus, antigenic distances, virus antigenicity prediction, antigenic drift, antigenic variants

## Abstract

**Introduction:**

Seasonal influenza A H3N2 viruses are constantly changing, reducing the effectiveness of existing vaccines. As a result, the World Health Organization (WHO) needs to frequently update the vaccine strains to match the antigenicity of emerged H3N2 variants. Traditional assessments of antigenicity rely on serological methods, which are both labor-intensive and time-consuming. Although numerous computational models aim to simplify antigenicity determination, they either lack a robust quantitative linkage between antigenicity and viral sequences or focus restrictively on selected features.

**Methods:**

Here, we propose a novel computational method to predict antigenic distances using multiple features, including not only viral sequence attributes but also integrating four distinct categories of features that significantly affect viral antigenicity in sequences.

**Results:**

This method exhibits low error in virus antigenicity prediction and achieves superior accuracy in discerning antigenic drift. Utilizing this method, we investigated the evolution process of the H3N2 influenza viruses and identified a total of 21 major antigenic clusters from 1968 to 2022.

**Discussion:**

Interestingly, our predicted antigenic map aligns closely with the antigenic map generated with serological data. Thus, our method is a promising tool for detecting antigenic variants and guiding the selection of vaccine candidates.

## 1 Introduction

**Figure 1:**
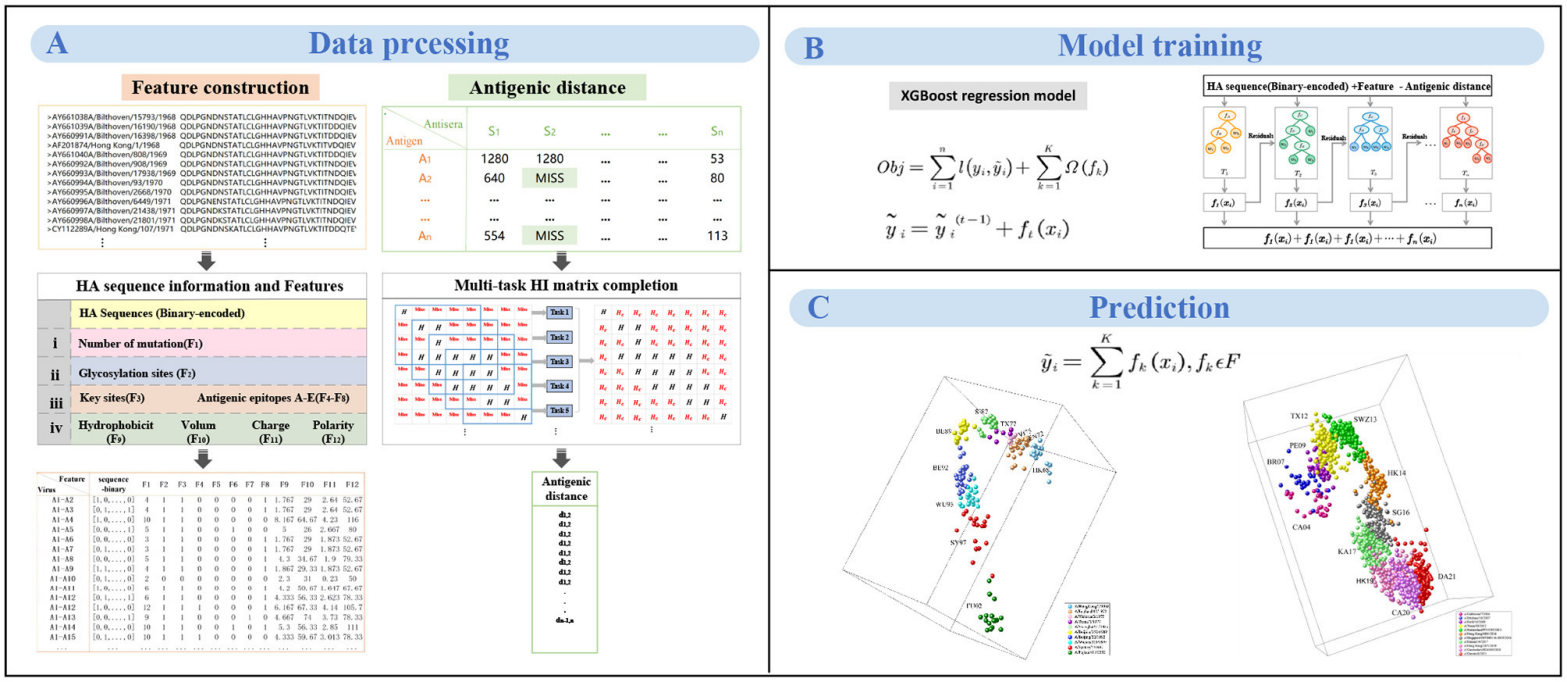
The workflow of MFPAD. **(A)** The dataset comprises two types of data: HA sequences and HI data. Four categories of features, and we introduce 12 features related to viral antigenicity. The antigenic distances are obtained through multi-task low-rank matrix completion of the HI matrix. **(B)** Establishing the quantitative relationship between virus sequences and antigenic distances using XGBoost regression model. **(C)** The antigenic evolution map of H3N2 influenza viruses from 1968 to 2022 includes two parts: (1) 270 HA sequences of viruses from 1968 to 2003, (2) 1,493 HA sequences of viruses from 2003 to 2022.

Each year, seasonal influenza results in an estimated 3–5 million cases of severe illness, culminating in roughly 290,000–650,000 respiratory-related deaths (Nelson and Holmes, 2007; Russell et al., 2008; Iuliano et al., 2018). The H3N2 influenza virus is one of the predominant subtypes responsible for these outbreaks. While vaccination remains the most effective measure to combat seasonal influenza, the perpetual evolution of influenza A viruses necessitates regular updates of the flu vaccines. Since the H3N2 viruses became prevalent in humans in 1968, they have spread worldwide and experienced significant antigenic evolution. This evolution is characterized by alternating phases: periods of relative stability are followed by phases of rapid phenotypic changes. Research conducted by Koel et al. (2013), as outlined in their antigenic cartography, reveals that from 1968 to 2003, ten distinct antigenic clusters of H3N2 viruses were identified. Moreover, data from the World Health Organization indicates that from 2003 to 2023, multiple additional antigenic clusters of H3N2 viruses have emerged (https://www.who.int/teams/global-influenza-programme/vaccines/who-recommendations/recommendations-for-influenza-vaccine-composition-archive). The surface glycoprotein Hemagglutinin(HA), responsible for attachment and fusion to host cell membranes, is the primary target of neutralizing antibodies (Webster et al., 1992; Wille and Holmes, 2020). Consequently, mutation(s) in the HA protein may trigger antigenic drift which allows the virus to evade the host's immune defenses, facilitating its continued spread and infection (Caton et al., 1982; Wilson and Cox, 1990; Peng et al., 2022b). At present, researchers largely depend on serological assays, such as the hemagglutination inhibition (HI) assay, to determine viral antigenicity. However, these techniques are labor-intensive, time-consuming, and offer only medium throughput (Sun et al., 2013). This underscores the pressing need to devise fast and precise methods to identify antigenic variants. Such advancements will optimize the selection of vaccines that align with emerging antigenic strains. With the advancements in sequencing technology and the concurrent accumulation of viral sequences and serological data in databases, efforts are being made to employ computational algorithms for the prediction of viral antigenicity (Kilbourne et al., 2002). Such methods have the potential to significantly enhance the detection of antigenic variants and optimize the selection of vaccine strains. For example, a statistical method to correlate the HI titer with the number of mutations in the HA sequence of viruses was developed by Lee and Chen (2004). Multiple and logistic regression were applied to assess the relationship between mutations in the HA sequence and HI data (Liao et al., 2008). Decision tree algorithms were applied to predict drift varients by extracting the association from HI data using information theory (Huang et al., 2009). While these methods lay the foundation for predicting the antigenic evolution of influenza viruses, they do not quantify the antigenic distance between the viruses. In recent years, there have been several attempts to establish a quantitative relationship between viral HA sequences and antigenic distance. For instance, Sparse learning methods have been proposed to identify key sites influencing antigenic changes and establish a quantitative relationship between key sites and antigenic distances (Sun et al., 2013; Yang et al., 2014). Previous research has already demonstrated that only a limited number of sites are active in the process of antigenic change (Smith et al., 2004). Regression models such as support vector regression and joint random forest regression were applied to establish a quantitative relationship between viral HA sequences and antigenic distances in order to identify drift variants (Ren et al., 2015; Yao et al., 2017). However, these methods only consider viral HA sequences, but biological experiments have unveiled many factors crucial to viral antigenicity, such as five primary antigenic regions, glycosylation of HA and so on. Some existing methods have already paid attention to the features related to antigenic change (Du et al., 2012; Han et al., 2019; Peng et al., 2022a), but the selected features either still are not associated with viral sequences or the number of selected features is limited and not comprehensive enough. Here, we propose a novel computational method, named MFPAD, that establishes a quantitative relationship between viral sequences and antigenic distances while integrating four categories of features influencing viral antigenicity. The overview of MFPAD is shown in Figure 1. MFPAD significantly improves the accuracy of identifying antigenic variants and reduces the prediction error of antigenic distance. We apply MFPAD to the H3N2 influenza A virus and successfully present its antigenic evolution patterns, and further confirm the positive impact of the four categories of features on prediction accuracy.

## 2 Materials and methods

### 2.1 HA sequence and serologic data

The HA sequences of H3N2 influenza viruses are collected from the Influenza Virus Database (https://www.ncbi.nlm.nih.gov/genomes/FLU/Database/nph-select.cgi?go=database). The serological data consists of two parts: (1) the first part contains cross-reactive HI antibody titers of 79 antisera with 270 H3N2 viruses isolated from 1968 to 2003, which is generated by Koel et al. (2013), (2) the second part contains cross-reactive HI titers of 173 serum with 1493 virus sequences isolated between 2003 and 2022 (collected from annuel and interim reports from Worldwide Influenza Centre Lab, https://www.crick.ac.uk/research/platforms-and-facilities/worldwide-influenza-centre/annual-and-interim-reports).

### 2.2 MFPAD model

Based on the integration of information from the literature, we select four categories of features based on HA sequence that are closely related to antigenic change. We then develop a novel computational framework integrating the HA sequence information with 12 features and propose a model to quantitatively correlate HA sequences with antigenic distance, thereby improving the accuracy of antigenic variant recognition and reducing the prediction error of antigenic distance. The computational framework consists of three steps: feature representation based on HA sequences, multi-task HI matrix completion for antigenic distances, training and prediction of the XGBoost model.

#### 2.2.1 Feature representation based on HA sequences

A pair of HA sequences (only HA1 sequences were used) is generally represented in two ways: one is binary representation, and the other is Pattern-Induced Multi-sequence Alignment (PIMA) scoring function (Smith and Smmith, 1992). Through comparison (see in Section 3.3), the binary method yields higher prediction accuracy, because of which we converted the HA sequence into the binary format. Based on features identified by various researchers that affect the antigenicity of influenza A viruses, a total of four categories of features are selected in this study. We calculate four categories of features between each pair of viruses based on the HA sequence. The primary category encompasses the number of substitutions in the viral HA sequences (Feature 1), since researchers have demonstrated that the number of mutations will substantially change the viral antigenicity (Lee and Chen, 2004). The second group pertains to the glycosylation sites (Feature 2), which was identified as the key factor affecting viral antigenicity (Wang et al., 2010; Tate et al., 2014; Hervé et al., 2015; Abdelwhab et al., 2016; Gu et al., 2019; York et al., 2019; Gao et al., 2021; Yin et al., 2021; Xu et al., 2022). The tertiary group integrates crucial antigenic positions (Feature 3) and substitutions within the five predominant antigenic regions designated as A, B, C, D, and E (Feature 4-8). Because many researchers have mapped the antigenic epitopes of H3N2 viruses and demonstrated that the viral antigenicity was mainly determined by five antigenic regions in the globular head of HA (Wiley et al., 1981; Tsuchiya et al., 2001; Hensley et al., 2009). More recently, Koel et al. and others further identified that key substitutions near the receptor-binding site in HA mainly determine the antigenic evolution of H3N2 viruses (Gerhard et al., 1981; Koel et al., 2013; Kong et al., 2021). The quaternary category embodies four intrinsic physicochemical properties of amino acids: hydrophobicity (Feature 9), volume (Feature 10), charge (Feature 11), and polarity (Feature 12), which significantly determine the antibody-protein interactions (Karadag et al., 2020). For a given pair of viruses *i*, feature *j*(*j* = 1, ..., *N, N* = 12), *N* represents the number of features. *f*_*ij*_ represents feature *j* for a pair of virus *i*. For Feature 1, *f*_*ij*_ is calculated as the amino acid site mutations in the HA sequence of virus pair *i*. For Feature 2, we identify glycosylation sites in the HA sequence based on the NXT/S sequons (where X is any amino acid except proline), and then compare whether these sequons exist in the pair of virus *i*. If there is a glycosylation sequon, then *f*_*ik*_ = 1, otherwise *f*_*ik*_ = 0. For features 3-8, the calculation method for *f*_*ij*_ is similar to the calculation method for the Feature 2. If there is a mutation occurring at any site within the set, then *f*_*ij*_ = 1, otherwise *f*_*ij*_ = 0. For features 9-12, if the number of amino acid mutations between virus pairs is less than 3, then *f*_*ij*_ is calculated as the mean difference in the quantitative values of the physicochemical properties of the mutation sites. If the number of mutations is greater than 3, only the 3 amino acid sites with the greatest differences in quantitative values are considered.

#### 2.2.2 Multi-task HI matrix completion for antigen distances

The antigenic distances between viruses are used to measure the degree of antigenic difference between viruses. The antigenic difference is typically assessed and measured through HI assay. The HI matrix typically presents three different types of data: high-reacting values, low-reacting values, and missing values (Sun et al., 2013). After arranging the antigens and antisera in increasing order by year, the data within the HI matrix exhibits an overall banded distribution. The diagonal region primarily consists of high-reacting values and missing values, while others consist of low-reacting values and missing values. The completion of missing values in the HI matrix can be transformed into a low-rank matrix completion problem (Cai et al., 2010). To minimize the impact of low-reacting values, the completion task of the HI matrix is divided into multiple subtasks using a time sliding window (Cai et al., 2010; Sun et al., 2013). Due to the division of the task and the diversity of subsets, the amount of data and information involved varies from each subtask, resulting in different optimal ranks for each subtask. Nuclear norm regularization techniques can address the challenge of optimizing the general rank for all tasks (Jaggi et al., 2010; Han and Zhang, 2016). Formally, given a *m* x *n* matrix A, The matrix completion problem infers missing values and replaces low-reacting values with more confident values by solving the following optimization problem:


(1) $$\min\frac{1}{2}\sum_{i=1}^{m}\sum_{i=1}^{n}\left(H_{i,j}^{\Omega }-A_{i,j}^{\Omega })(H_{i,j}^{\Omega }>\theta \right)+\lambda ||H|{|}_{*}$$

(2) $$\begin{array}{l} ||H|{|}_{*}=\overset{\min\left(m,n\right)}{\underset{i=1}{\sum }}\sigma_{i} \end{array}$$

where the matrix *H* is the estimate of *A*, Ω is a set of low-reacting values and determined values. In equation (1), θ is denoted as the threshold for low-reacting values. In equation (2), ||*H*||_*_ is the nuclear norm, which is the sum of all singular values of the matrix *H*. And λ is a regularization parameter that strikes a compromise between data fitting and matrix rank regularization.

The antigenic distances between viruses are derived from HI matrix (Cai et al., 2010). Each unit of antigenic distance is equivalent to 2*log*_2_(*HI*). In the antigenic map, 2 units of the antigenic distance represent 4-fold change in HI titer. This threshold is also used as a criterion for assessing virus variants (Smith et al., 1999). If the antigenic distances between viruses are greater than 2 units, they are considered antigenically different and belong to different antigenic cluster. Conversely, they are deemed antigenically similar and belong to the same antigenic cluster.

#### 2.2.3 Training and prediction of the XGBoost model

The overall goal of this study is to develop an HA sequence based method to predict viral antigenicity. We employ the XGBoost regression model to establish a quantitative relationship between viral sequences and antigenic distances. XGBoost is a machine learning model based on ensemble principles and its core idea root in gradient boosting and decision trees (Chen and Guestrin, 2016). For a given virus pair *i*, the feature vector is represented as *x*_*i*_, and *y*_*i*_ represents its antigenic distance. The objective function of the XGBoost model is defined as follows:


(3) $$\begin{array}{l} Obj^{\left(t\right)}=\overset{n}{\underset{i=1}{\sum }}l\left(y_{i},{\hat{y_{i}}}^{\left(t\right)}\right)+\Omega \left(f_{t}\right)+C \end{array}$$

where *i* represents a pair of virus, *n* denotes the total number of virus pairs, $l\left(y_{i},{\hat{y_{i}}}^{\left(t\right)}\right)$ is the loss function, ${\hat{y_{i}}}^{\left(t\right)}$ represents the current prediction, and *y*_*i*_ represents the true value. In equation (3), Ω(*f*_*t*_) represents the complexity of the t-th tree, and C represents a constant term. The prediction result for the t-th round is obtained by summing the prediction results of the previous subtrees as illustrated in equation (4):


(4) $$\begin{array}{l} {\hat{y_{i}}}^{\left(t\right)}={\hat{y_{i}}}^{\left(t-1\right)}+f_{t}\left(x_{i}\right) \end{array}$$

${\hat{y_{i}}}^{\left(t-1\right)}$ represents the model predictions from the previous *t*−1 rounds, and *f*_*t*_(*x*_*i*_) represents the tree. The loss function use mean squared error:


(5) $$\begin{array}{l} l\left(y_{i},{\hat{y_{i}}}^{\left(t\right)}\right)=\frac{1}{n}\overset{n}{\underset{i=1}{\sum }}{\left(y_{i}-{\hat{y_{i}}}^{\left(t\right)}\right)}^{2} \end{array}$$

Ω(*f*_*t*_) represents the complexity of the t-th tree in equation (5), where complexity is defined as the sum of the number of leaf nodes and the square sum of the weights of all leaf nodes:


(6) $$\begin{array}{l} \Omega \left(f_{t}\right)=\gamma T+\frac{1}{2}\lambda \overset{T}{\underset{j=1}{\sum }}w_{j}^{2} \end{array}$$

*T* represents the number of leaf nodes, γ denotes the difficulty of node splitting, λ is used to indicate the sparsity of L2 regularization, and *w_j_* represents the weight of the leaf node *j* in equation (6).

## 3 Results

### 3.1 MFPAD predicts the antigenic evolution of H3N2 influenza viruses

**Figure 2:**
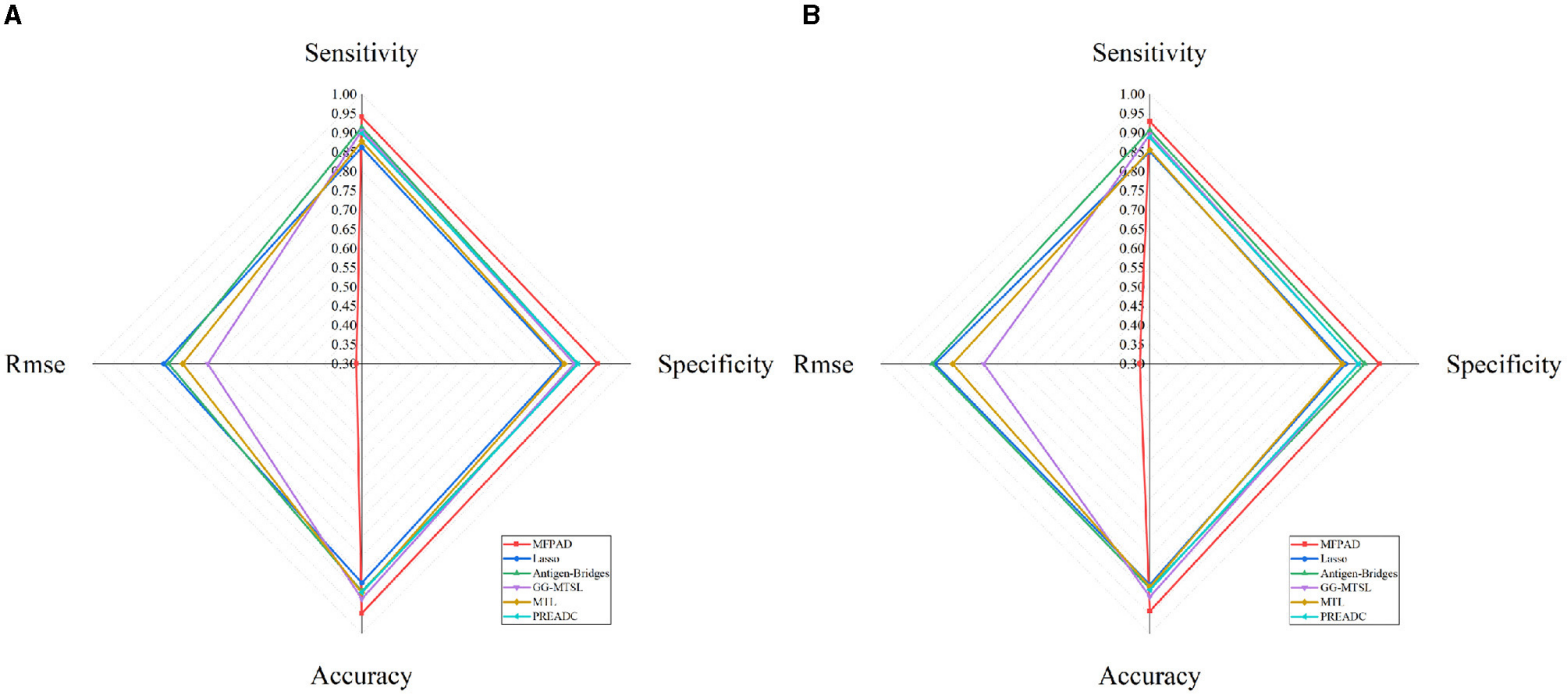
Comparative Radar Chart of Model Performance Metrics. **(A)** Represents the performance metrics of models on the dataset from 1968 to 2003. **(B)** Represents the performance metrics of models on the dataset from 2003 to 2022.

By combining HA sequences of the influenza viruses with 12 relevant features affecting viral antigenicity, we establish a quantitative relationship with viral HA sequences and antigenic distances, thereby improving the accuracy of assessing viral antigenicity. The MFPAD is applied to gain insight into the antigenic evolution of H3N2 viruses. After collecting available cross-reactive HI data between H3N2 viruses and sera, we process the missing values in HI data by using a multi-task low-rank matrix completion method. In addition to using the binary representation of the viral HA sequence, we additionally select four categories of features, including a total of 12 features closely related to viral antigenicity. These features encompass the number of mutations, glycosylation sites, key antigenic positions and mutations in five antigenic regions, and four kinds of amino acid physicochemical properties (hydrophobicity, polarity, charge, volume). We utilize the XGBoost regression model to establish a quantitative relationship between viral sequences and antigenic distances. Ultimately, we apply MFPAD to H3N2 virus sequences to predict the antigenic evolution process of H3N2 influenza viruses. In order to obtain the completion matrix with the smallest error, we choose a parameter λ = 0.3, and set the size of the time sliding window to 12 during the multi-task low-rank matrix completion process (Cai et al., 2010; Sun et al., 2013). Error calculation is carried out by randomly selecting 10% of the high-response values in the matrix for testing. During the building of the XGBoost regression model, we employ a stratified sampling method to select 90 virus sequences from virus isolated from 1968 to 2003 and 502 viruses sequences from viruses isolated from 2003 to 2022 as training data. The antigenic evolution map includes all the viruses in the dataset. The model performance evaluation is carried out using ten-fold cross-validation, and model hyperparameters are optimized using grid search. The selected parameters are as follows: booster type is based on tree models, maximum tree depth is set to 7, learning rate is 0.1, the number of decision trees is 200, the minimum loss reduction for tree growth is 0.1, and the remaining parameters are default values. The model achieves a Root Mean Square Error (RMSE) of 0.314 in the virus dataset from 1968 to 2003 and 0.326 in the virus dataset from 2003 to 2022. When the antigenic distances between viruses exceeded 2, it is considered that the antigenic drift occurred. Based on this, 2 units distance are set as the threshold for antigenic variants. MFPAD yields a high prediction accuracy of 0.948 in the virus dataset from 1968 to 2003 and 0.942 in the virus dataset from 2003 to 2022 for identifying virus variants. Furthermore, we utilize historical training data from 1968 to the target prediction year to assess our model's predictive accuracy for upcoming seasons. The average accuracy in predicting antigenic variants emerging in the coming year reaches 92.3%. Detailed information can be found in Supplementary Tables S2, S3. To evaluate the accuracy of MFPAD, the MFPAD is compared with other models, including two single-task models [Lasso (Cai et al., 2012) and Antigen-Bridges (Sun et al., 2013)], two multi-task models [GG-MTSL (Han et al., 2019) and MTL (Liu et al., 2012)], and the PREDAC model based on network built on virus similarity (Du et al., 2012). Among the compared models, MFPAD exhibit the smallest error and the highest accuracy in predicting antigenic variants, as shown in Table 1 and Figure 2. These results indicate that the quantitative relationship established in this study is significantly effective and accurate in predicting antigenic distance between viruses and identifying antigenic variants.

**Table 1:** Model performance comparison.

**Model**	**Accuracy**		**RMSE**		**Sensitivity**		**Specificity**	
	**1968–2003**	**2003–2022**	**1968–2003**	**2003–2022**	**1968–2003**	**2003–2022**	**1968–2003**	**2003–2022**
MFPAD	0.948	0.942	0.314	0.326	0.941	0.929	0.913	0.903
Lasso	0.869	0.873	0.815	0.859	0.862	0.851	0.822	0.809
Antigen-Bridges	0.891	0.883	0.803	0.866	0.914	0.907	0.861	0.859
GG-MTSL	0.911	0.905	0.701	0.732	0.906	0.895	0.853	0.842
MTL	0.895	0.879	0.766	0.813	0.878	0.855	0.827	0.801
PREADC	0.894	0.887	N/A	N/A	0.898	0.887	0.863	0.843

Performance of MFPAD, two single-task models (Lasso and Antigen-Bridges), two multi-task models (GG-MTSL and MTL), and the PREDAC method. Root Mean Square Error (RMSE) represents the error in predicting antigenic distances, accuracy represents the performance of the model in identifying antigenic variants with a threshold of 2.

### 3.2 Antigenic evolution of H3N2 influenza viruses

**Figure 3:**
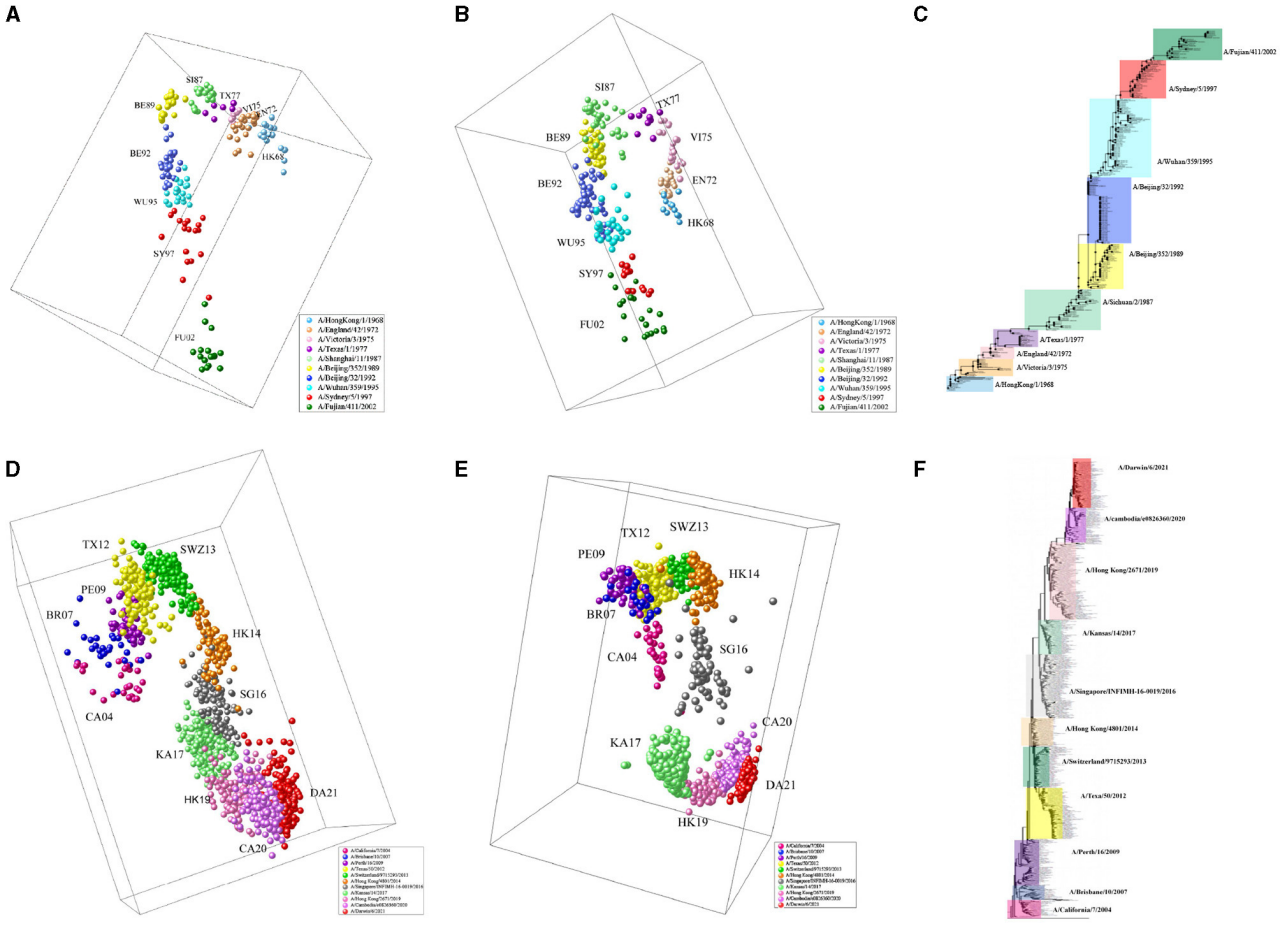
The antigenic evolution map of H3N2 influenza viruses from 1968 to 2022. **(A, D)** Represent antigenic evolution maps based on viral sequences, while **(B, E)** represent those based on HI data. **(C, F)** Represent phylogenetic trees for the periods 1968-2003 and 2003-2022.

We apply MFPAD to H3N2 influenza viruses, which includes (1) 270 HA sequences of virus from 1968 to 2003 and (2) 1,493 HA sequences of virus from 2003 to 2022. We predict their antigenic distances and create antigenic evolution maps through Multidimensional Scaling(MDS). Through the antigenic evolution maps, we are able to identify major antigenic clusters and pathways of antigenic evolution, gaining insights into the antigenic distances among different virus strains. We identify a total of 10 virus antigenic clusters for the period from 1968 to 2003 (HK68, EN72, VI75, TX77, SI87, BE89, BE92, WU95, SY97, FU02), as well as 11 virus antigenic clusters for the period from 2003 to 2022 (CA04, BR07, PE09, TX12, SWZ13, HK14, SG16, KA17, HK19, CA20, DA21), as shown in Figures 3A, D. To validate the accuracy and reliability of the antigenic evolution maps generated based on the computational method proposed in this study, we compare them with antigenic evolution maps constructed by using HI data measured with various antisera, as shown in Figures 3B, E. The results show that both maps exhibit similar evolutionary patterns, with each major predicted antigenic cluster matching that generated with serological data. Furthermore, We use RAxML to construct the phylogenetic tree of H3N2 viruses from 1968 to 2022, which is based on the Maximum Likelihood estimation method, as shown in Figures 3C, F. During the tree construction process, certain parameter choices are necessary. Specifically, we choose the General Time Reversible (GTR) substitution model and the Subtree Pruning and Regrafting (SPR) tree topology search algorithm. Additionally, RAxML supports Bootstrap analysis, and we performed 1,000 replicates for the analysis. Our results exhibit consistency and similarity when compared to the evolutionary patterns presented in phylogenetic trees, thus validating the accuracy and reliability of our predicted results.

### 3.3 The impact of 12 features on model performance

**Figure 4:**
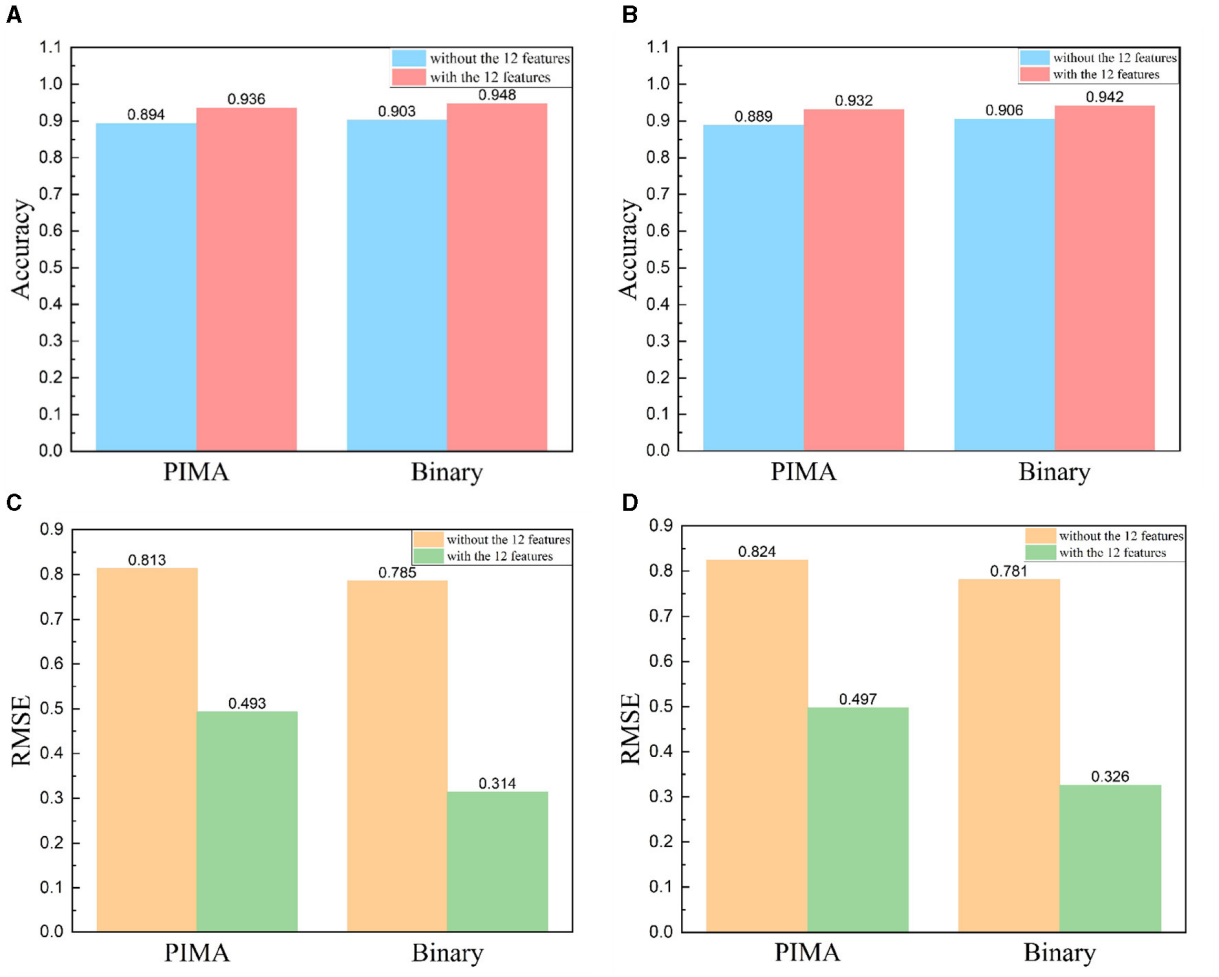
Comparative results with and without the addition of 12 features, HA sequence represented using PIMA or binary encoding. **(A, B)** Includes viruses from 1968 to 2003 and **(C, D)** includes viruses from 2003 to 2022. **(A, C)** Comparison results of accuracy, **(B, D)** comparison results of RMSE.

In antigenic evolution studies of influenza viruses, viral sequences are commonly depicted using binary encoding or PIMA to quantify the genetic or antigenic distances between viruses. In this study, we investigate beyond solely considering virus sequences. Instead, we incorporate 12 features intrinsically linked to the antigenic evolution of influenza A viruses (Gerhard et al., 1981; Koel et al., 2013; York et al., 2019). Originating from viral HA sequences, these features are grouped into four categories. The first encompasses the number of amino acid mutations within the virus HA sequences. The second category of features include the glycosylation sites. The third category of features include the key antigenic positions, and the mutations in the five antigenic regions (A, B, C, D, and E). The fourth set of features focus on the physicochemical attributes of amino acids (hydrophobicity, volume, charge, and polarity). To substantiate the impact of these 12 features on enhancing the accuracy and reliability of antigenic evolution prediction, we undertake comparative tests. We conduct a comparative analysis between two methods: the first method involves training an XGBoost model utilizing solely the genetic distances represented by viral HA sequences, characterized through PIMA or binary encoding as features; the second method incorporates an additional 12 features into the model. Our experimental results demonstrate that the enriched model, supplemented with these 12 features, substantially outperform the model relying solely on PIMA or binary-encoded HA sequences. Notably, the inclusion of these 12 features markedly enhances the accuracy of predicting antigenic variants and also leads to a reduction in RMSE, as shown in Figure 4.

### 3.4 Changes in the 12 features during antigenic drift

**Figure 5:**
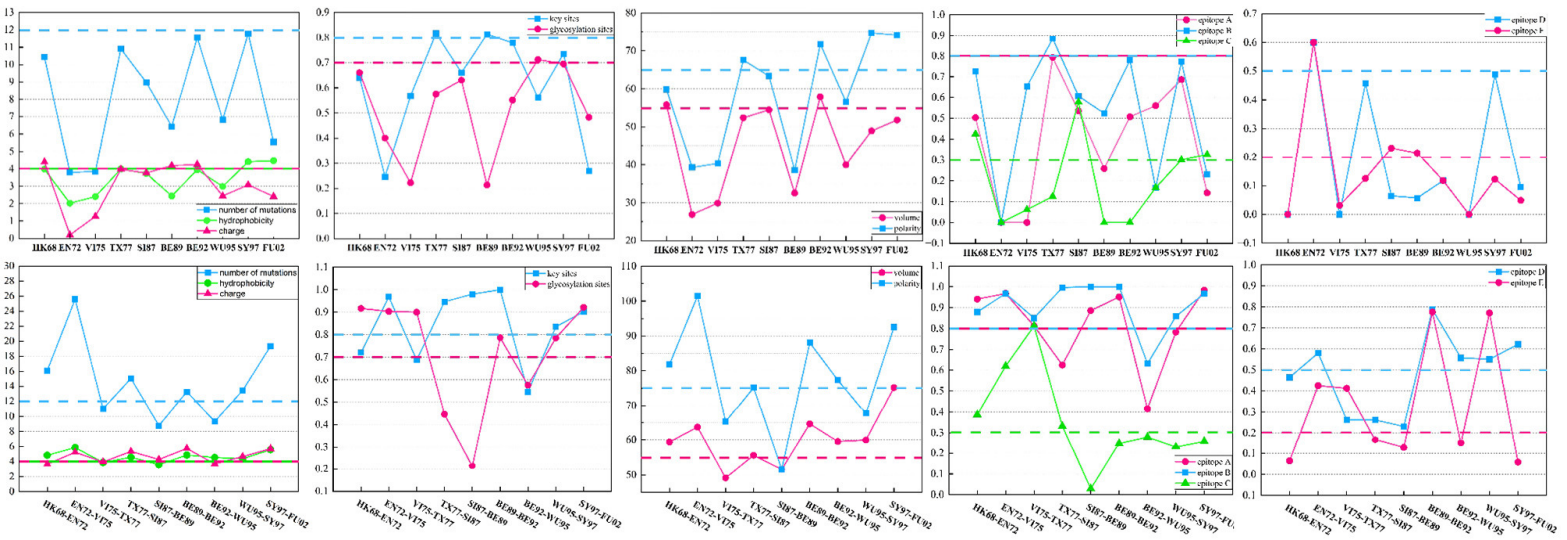
The differences in the 12 features between viruses within two adjacent antigenic clusters during antigenic drift and among viruses within the same antigenic cluster. Twelve features between viruses from two antigenic clusters show varying degrees of increase compared to the values between viruses within the same antigenic cluster.

To probe the significance of these supplementary features in the antigenic evolution of the H3N2 influenza viruses, we calculate the mean values of these 12 features between two adjacent antigenic clusters undergoing antigenic drift as well as within individual antigenic clusters. Compared to values derived from the individual antigenic clusters, the values of these features derived from two adjacent antigenic clusters display various levels of increase, as shown in Figure 5. The accumulation of mutations leads to a higher probability of generating antigenic variants. In the virus dataset from 1968 to 2003, within one antigenic cluster, the value for virus mutations averages below 12, while in 66.7% of adjacent clusters, the value for virus mutations averages above 12. Glycosylation can either shield or reveal specific antigenic epitopes on HA proteins, thus plays an important role on viral antigenicity (York et al., 2019). A similar trend is observed for glycosylation sites, with 90% of individual antigenic clusters having mutation frequencies below 0.7, but in 66.7% of adjacent clusters, these frequencies are above 0.7. Studies have identified that there were five main antigenic regions (A-E) in HA (Wiley et al., 1981; Wilson et al., 1981), and the antigenicity of H3N2 viruses is mainly determined by key antigenic positions around the receptor binding site in HA (Koel et al., 2013, 2014). In 80% of individual antigenic clusters, virus mutations at key antigenic positions remain below 0.8, while in 66.7% of neighboring clusters, they consistently exceed 0.8. Furthermore, mutations in the five major antigenic regions of the H3N2 viruses are closely associated with changes in antigenicity. In individual antigenic clusters, the virus mutation frequencies in antigenic region A are consistently below 0.8, while 66.7% of adjacent antigenic clusters have frequencies exceeding 0.8. For antigenic region B, 90% of viruses within antigenic clusters have mutation frequencies below 0.8, while in adjacent clusters, 88.9% are above 0.8, with 55.6% surpassing 0.9. Regarding antigenic region C, 60% of viruses within antigenic clusters have mutation frequencies below 0.3, while 44.4% of adjacent antigenic clusters have frequencies above 0.3. As for antigenic region D, in 90% of antigenic clusters, virus mutation frequencies are below 0.5, while 55.7% of adjacent antigenic clusters have frequencies above 0.5. Finally, for antigenic region E, in 70% of antigenic clusters, virus mutation frequencies are below 0.2, while in 44.4% of adjacent antigenic clusters, they exceed 0.2. Overall, the mutation frequencies in antigenic regions C and E, as well as their variation within individual and adjacent antigenic clusters, are slightly lower than those in regions A, B, and D. In addition, we also examine four amino acid physicochemical properties (hydrophobicity, polarity, charge, and volume), which significantly determine the antibody-protein interactions (Karadag et al., 2020). In 80% of antigenic clusters, the differences in hydrophobicity between viruses are below 4, while in 77.8% of adjacent antigenic clusters, the differences in hydrophobicity are above 4. Regarding changes in amino acid volume, 80% of individual antigenic clusters remain below 55, while 77.8% of adjacent antigenic clusters exceed 55. Differences in amino acid charge are below 4 in 70% of individual antigenic clusters, but in 66.7% of adjacent antigenic clusters, they are above 4. Within individual antigenic clusters, differences in polarity between viruses remain below 75, while in 66.7% of adjacent antigenic clusters, they exceed 75. The analysis of the virus from 2003 to 2022 is presented in the Supplementary material. Compared to viruses within individual antigenic clusters, viruses within the two antigenic clusters show varying degrees of increased differences in these four amino acid physicochemical properties. Changes in the physicochemical properties of amino acids can affect the viral antigenicity and its interaction with the host's immune response.

### 3.5 Inferring the antigenicity of H3N2v variants based on virus sequences

**Figure 6:**
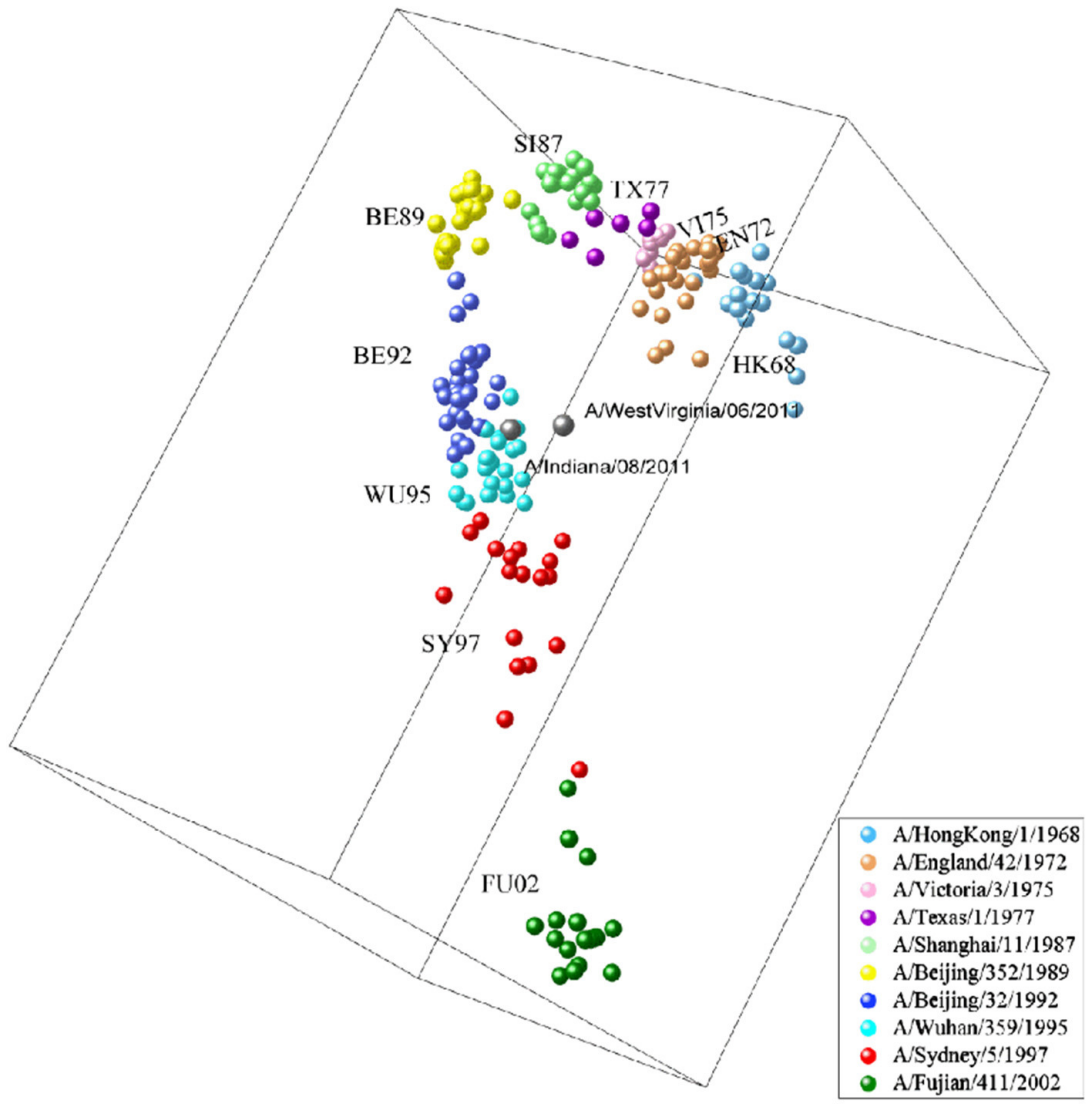
H3N2v-like virus (A/Indiana/08/2011, A/WestVirginia/06/2011) marked in cyan, an antigenic variant that emerged in 2011 with antigenic similarity to BE92 and WU95.

Based on our proposed computational method, it is possible to make an initial assessment of the antigenicity of influenza viruses without biological experiments. H3N2v virus is a variant of the H3N2 influenza virus, and was first discovered at the United States Agricultural Fair in 2011 [Centers for Disease Control and Prevention (CDC), 2012]. During the period from August 2011 to April 2012, there were a total of 2,055 reported cases of H3N2v virus infections (Biggerstaff et al., 2013). Through the analysis of the antigenic evolution map of H3N2 virus, we find that the strains of H3N2v (A/Indiana/08/2011 and A/WestVirginia/06/2011) exhibit the closest antigenic distance to the BE92 (A/Beijing/32/1992) and WU95 (A/Wuhan/359/1995) virus strains, as shown in Figure 6. This indicates their similarity in viral antigenicity, and this research finding has been previously confirmed in earlier studies (Sun et al., 2013). We compare the virus sequences of H3N2v (A/Indiana/08/2011, A/WestVirginia/06/2011) with WU95 (A/Wuhan/359/1995) and BE92 (A/Beijing/32/1992), and find that their sequence similarities are 88.1% and 89.6%, respectively. The H3N2v virus is believed to transmit from humans to pigs during the 1990s and humans come into contact with pigs infected with the H3N2v virus in 2011, leading to its reemergence in the human (Feng et al., 2013). Such inter-species transmission events could have occurred in settings like farms, swine-rearing facilities, or other environments with close interactions between humans and pigs.

## 4 Discussion

In this study, We propose a novel method for quantifying antigenic distances based on viral sequences. MFPAD not only takes into account the viral sequences but also integrates four categories of features related to antigenic change. Compared to previously established methods for predicting antigenic distances between viruses, MFPAD exhibits smaller errors in predicting antigenic distances between viruses and demonstrates higher accuracy in identifying antigenic variants.

With the development of high-throughput sequencing technologies, sequence data has become both cost-effective and rapid to obtain. Compared to hemagglutination inhibition (HI) data, sequence data is also more reliable and less susceptible to laboratory-specific variations. In this context, accurate computation of antigenic distances between viruses based on HA sequences has become critically important for virus classification, epidemiological investigation, and vaccine design. However, existing methods still have certain errors and limitations. Currently, research on the evolution of influenza viruses primarily focuses on viral sequence features, with a specific emphasis on amino acid mutation sites. For instance, applying regression models to construct HA sequences or identify key sites in order to establish a quantitative relationship with antigenic distances (Sun et al., 2013; Ren et al., 2015; Yao et al., 2017; Han et al., 2019). Nevertheless, besides mutations in amino acid positions within viral sequences, there are multifaceted factors influencing antigenic evolution. Li et al. (2020) treat glycosylation sites as a separate category of features. Additionally, in the approach proposed by Du et al. (2012), features include various physicochemical properties of amino acids. These properties directly influence the structure, function, and stability of viral proteins. In this study, We integrate the viral sequence information along with categories of features related to antigenic change. These features include the number of sequence mutations, glycosylation sites, key antigenic positions, five major antigenic regions, and four physicochemical properties of amino acids, which encompass hydrophobicity, volume, charge, and polarity. By comprehensively considering these features, we can conduct a more comprehensive assessment of viral antigenic change, improve the accuracy of viral variant recognition, and reduce the error in predicting antigenic distances. When new antigenic variants emerge, MFPAD enables a rapid assessment of their antigenicity, determining their potential for epidemiological relevance and transmission risk. It can also provide essential guidance for vaccine preparation.

The 12 features in this study are closely associated with the antigenic evolution of H3N2 influenza virus. Among viruses from adjacent antigenic clusters, these 12 features exhibit varying degrees of increase in their values when compared to viruses within the same antigenic cluster. We observe that the frequency of mutations occurring in antigenic positons A, B, and D is higher in neighboring antigenic clusters compared to epitopes C and E. Particularly in antigenic epitope B, in nearly 90% of antigenic drift events, the mutation frequency between viruses exceeds 0.8, with over 50% of antigenic drift events even surpassing 0.9. Ndifon et al. (2009) also highlight that amino acid mutations occurring in antigenic epitopes with high antigenic efficiency (A, B, and D) exhibit a stronger correlation with viral antigenic drift when compared to epitopes with low to moderate efficiency (C and E). Noteabley, biological studies have demenstrated that antigenic region A and B play an major role to induce host immune response, and antigenic region B is immunodomant (Popova et al., 2012; Broecker et al., 2018; Wu et al., 2020). The consistent between the predicted result and biological findings further demonstrate that our model is feasible to predict the antigenicity of H3N2 viruses.

In this study, we apply MFPAD to predict the antigenic evolution of H3N2 influenza viruses based on HA sequences, including 270 sequences spanning from 1968 to 2003, and an additional 1493 sequences from 2003 to 2022. Our predictive analyses reveal the existence of 21 distinct major antigenic clusters, aligning closely with those identified through biological experiments. Although MFPAD is a robust system, our ability to represent the antigenic evolution of H3N2 viruses is somewhat limited. Rather than presenting it as a single integrated antigenic map, we are constrained to depict it in two separate maps. This limitation arises from the challenge of training the XGBoost model using HI data obtained from two independent sources, yielding a crowded and less reliable predicted map (data not shown). Several issues contribute to the challenges in processing and interpreting the HI data. Firstly, the HI data originates from different laboratories and are measured using varying protocols. Secondly, recent H3N2 viruses exhibit reduced binding to red blood cells (RBCs), necessitating a shift from turkey RBCs to guinea pig RBCs for HI assays (Lin et al., 2012). Thirdly, the surface protein NA of recent H3N2 viruses display RBC agglutination activity, which could introduce further variability into the HI data (Lin et al., 2010; Mögling et al., 2017). For assessing the antigenicity of influenza viruses, the gold standard is typically the Microneutralization assay (MN) or Focus Reduction Assay (FRA). Therefore, generating more reliable biological data, such as MN and FRA results, to train the prediction model could further enhance the accuracy of our predictive model.

## Data availability statement

The original contributions presented in the study are included in the article/Supplementary material, further inquiries can be directed to the corresponding author.

## Author contributions

XL: Conceptualization, Data curation, Formal analysis, Methodology, Software, Validation, Writing – original draft, Writing – review & editing, Funding acquisition, Project administration, Resources, Supervision, Visualization. YL: Conceptualization, Data curation, Formal analysis, Methodology, Software, Validation, Writing – original draft, Writing – review & editing, Investigation. XS: Conceptualization, Data curation, Formal analysis, Methodology, Project administration, Resources, Supervision, Visualization, Writing – original draft. HK: Conceptualization, Data curation, Formal analysis, Funding acquisition, Methodology, Project administration, Resources, Supervision, Writing – original draft, Writing – review & editing.
