# Supplementary material for: A sequence-based machine learning model for predicting antigenic distance for H3N2 influenza virus
Source: Front Microbiol. 2024 Jan 19;15:1345794. doi: 10.3389/fmicb.2024.1345794 (PMC10834737; doi:10.3389/fmicb.2024.1345794)
Supplement: Supplementary file 1 [file Image_1.PDF]

# Supplementary Material

## 1 SUPPLEMENTARY TABLES AND FIGURES

### 1.1 Figures

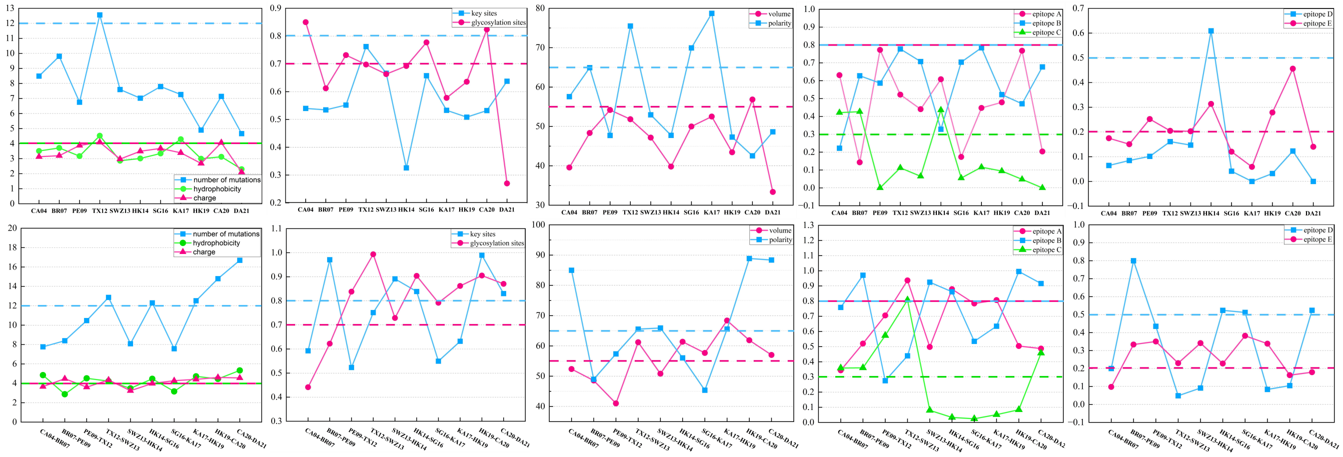


Figure S1: The differences in the 12 features between viruses within two adjacent antigenic clusters during antigenic drift and among viruses within the same antigenic cluster from 2003 to 2022. 12 features between viruses from two antigenic clusters undergoing antigenic drift show varying degrees of increase compared to the values between viruses within the same antigenic cluster.
