# Supplementary material for: A sequence-based machine learning model for predicting antigenic distance for H3N2 influenza virus
Source: Front Microbiol. 2024 Jan 19;15:1345794. doi: 10.3389/fmicb.2024.1345794 (PMC10834737; doi:10.3389/fmicb.2024.1345794)
Supplement: Supplementary file 2 [file Table_1.PDF]

frontiers

Supplementary Material

1 SUPPLEMENTARY TABLES AND FIGURES

1.1 Tables

Table S1. First 50 key amino acid positions

Site	Importance	Site	Importance
276	0.19814664	144	0.002218326
57	0.10043421	135	0.002185147
193	0.0208132	124	0.0021339213
189	0.01423177	196	0.0020576264
244	0.00894378	213	0.0020533479
217	0.00728464	194	0.0020046113
126	0.00591782	199	0.0017301932
142	0.00571582	156	0.0017205095
121	0.00428428	129	0.0012120534
155	0.00420144	299	0.0012016776
133	0.0040563825	59	0.0012016776
94	0.0040484667	230	0.0011664198
116	0.0036839999	158	0.0011550115
157	0.0034272354	242	0.001142261
233	0.0031516682	63	0.0010087916
92	0.0029276279	75	0.0009678056
197	0.0028733283	163	0.00092410715
260	0.0027578375	29	0.0009153476
262	0.0027317326	164	0.00088667887
67	0.0026441447	271	0.00087875314
307	0.0025975644	190	0.0008124144
131	0.002422432	278	0.00081103697
83	0.002364283	5	0.00078988523
54	0.002351441	82	0.0007882415
214	0.00224637	21	0.0007845584

Table S2. The predictive accuracy for pred1, pred2, and pred3

Train	Pred1	Pred2	Pred3
[1968,2003]	0.918	0.889	0.854
[1968,2004]	0.924	0.891	0.848
[1968,2005]	0.915	0.893	0.864
[1968,2006]	0.918	0.893	0.842
[1968,2007]	0.926	0.897	0.869
[1968,2008]	0.935	0.892	0.846
[1968,2009]	0.914	0.899	0.856
[1968,2010]	0.920	0.888	0.869
[1968,2011]	0.915	0.883	0.854
[1968,2012]	0.933	0.885	0.855
[1968,2013]	0.924	0.897	0.865
[1968,2014]	0.937	0.893	0.849
[1968,2015]	0.919	0.896	0.862
[1968,2016]	0.924	0.895	0.854
[1968,2017]	0.916	0.891	0.844
[1968,2018]	0.923	0.888	0.855
[1968,2019]	0.916	0.898	0.867
[1968,2020]	0.910	0.889	0.854
[1968,2021]	0.933	0.883	—
[1968,2022]	0.937	—	—

Note: ”—” indicates that until now(2023), there is no test set available .

Table S3. The average predictive accuracy for pred1, pred2, and pred3

Period	Pred1	Pred2	Pred3
Average	0.923	0.892	0.856
